# Frameshift Variant in Novel Adenosine-A1-Receptor Homolog Associated With Bovine Spastic Syndrome/Late-Onset Bovine Spastic Paresis in Holstein Sires

**DOI:** 10.3389/fgene.2020.591794

**Published:** 2020-11-30

**Authors:** Frederik Krull, Marc Hirschfeld, Wilhelm Ewald Wemheuer, Bertram Brenig

**Affiliations:** Department of Animal Sciences, Faculty of Agricultural Sciences, Institute of Veterinary Medicine, University of Göttingen, Göttingen, Germany

**Keywords:** bovine spastic syndrome, late-onset bovine spastic paresis, hereditary spastic paraplegia, Parkinsion's disease, LOC100848076, adenosine-A1-receptor

## Abstract

Since their first description almost 100 years ago, bovine spastic paresis (BSP) and bovine spastic syndrome (BSS) are assumed to be inherited neuronal-progressive diseases in cattle. Affected animals are characterized by (frequent) spasms primarily located in the hind limbs, accompanied by severe pain symptoms and reduced vigor, thus initiating premature slaughter or euthanasia. Due to the late onset of BSP and BSS and the massively decreased lifespan of modern cattle, the importance of these diseases is underestimated. In the present study, BSP/BSS-affected German Holstein breeding sires from artificial insemination centers were collected and pedigree analysis, genome-wide association studies, whole genome resequencing, protein–protein interaction network analysis, and protein-homology modeling were performed to elucidate the genetic background. The analysis of 46 affected and 213 control cattle revealed four significantly associated positions on chromosome 15 (BTA15), i.e., AC_000172.1:g.83465449A>G (–log_10_P = 19.17), AC_000172.1:g.81871849C>T (–log_10_P = 8.31), AC_000172.1:g.81872621A>T (–log_10_P = 6.81), and AC_000172.1:g.81872661G>C (–log_10_P = 6.42). Two additional loci were significantly associated located on BTA8 and BTA19, i.e., AC_000165.1:g.71177788T>C and AC_000176.1:g.30140977T>G, respectively. Whole genome resequencing of five affected individuals and six unaffected relatives (two fathers, two mothers, a half sibling, and a full sibling) belonging to three different not directly related families was performed. After filtering, a homozygous loss of function variant was identified in the affected cattle, causing a frameshift in the so far unknown gene locus LOC100848076 encoding an adenosine-A1-receptor homolog. An allele frequency of the variant of 0.74 was determined in 3,093 samples of the 1000 Bull Genomes Project.

## Introduction

Bovine spastic syndrome (BSS) is a cramp-causing disease in cattle, which is commonly assumed to be a hereditary pathology of the central nervous system (CNS) since its first description in 1941 (Frauchiger and Hofmann, 1941; Goeckmann et al., 2018). Usually one or both hind limbs are affected and show intermediating clonic and tonic spasms. Rarely, other regions like back muscles or even the entire body are affected. Kyphosis and weight loss are often seen as ancillary effects (Goeckmann et al., 2018). Seizures can last a few seconds to several minutes and then vanish for a time or quickly reappear. The disease proceeds chronic-progressively and usually ends up in slaughter of the affected animal or immobility and euthanasia. BSS is reported to appear (more) frequently in animals with straight hocks (Roberts, 1953).

No curative treatment is available so far. Only palliative therapy options like analgesia or the application of glucocorticoids and tranquilizers have been described; however, the long-term prognosis of BSS is very bad/infaust (Gentile and Testoni, 2006). Adaption of the animal's environment is also a therapeutic tool. Muscle waste at the spasmic region is a common sign. In general, BSS starts with mild symptoms with average onset after the beginning of the reproduction period of cattle. Frequently, secondary claw diseases contribute to BSS (conspicuousness) because seizures are assumed to cause severe pain. Therefore, BSS is also a highly relevant animal welfare issue. To reduce the risk of hereditary BSS, affected animals should be excluded from breeding.

The majority of symptomatic animals is aged 3–7 years. Higher incidence is assumed for male cattle with an increasing prevalence of BSS within the last five decades (De Vlamynck et al., 2014; Goeckmann et al., 2016). There are clinical reports of BSS in Holstein and at least five other breeds, but BSS occurrence was expected in all cattle breeds already in the early 1950s (Roberts, 1953; Goeckmann et al., 2018).

Differential diagnosis for BSS is difficult. First, symptoms can originate from suboptimal husbandry conditions (e.g., hypocalcemia, claw disorders) and therefore offer curative treatment options. Second, symptoms can also indicate the late-onset form of bovine spastic paresis (BSP) (Goeckmann et al., 2016). BSS and BSP could also be two different forms of the same genetic syndrome. BSP was first reported in 1922 as isolated contraction of the gastrocnemius muscle and later also categorized as a central nervous syndrome (Hamoir, 1922; Goeckmann et al., 2016). A very frequently used sire (born in 1998) is suspicious to pass on BSP.

BSS seems to be an emerging problem predominantly occurring in high-yield sires at artificial insemination centers during the past two decades, thus most likely spreading within the current Holstein population (Gentile and Testoni, 2006). The mode of inheritance remains unclear. Currently, four hypotheses on genetic transmission are discussed, i.e., non-autosomal, autosomal recessive, autosomal dominant, or multigenic; however, all of these have incomplete penetrance (Goeckmann et al., 2018). Recently, a genome-wide association study in the North American Holstein population showed associations of BSS on bovine chromosomes 7 and 9 and therefore support the hypothesis of an autosomal inheritance (Neustaeter et al., 2015). Estimates of the disease prevalence vary but are usually lower than 5% (Neustaeter et al., 2015; Goeckmann et al., 2016, 2018). An increasing degree of inbreeding might exert a major influence on BSS/late-onset BSP prevalence in the future (Gentile and Testoni, 2006).

Already in 1948, BSS was compared with human multiple sclerosis due to the clinical signs and the age of onset (Evers, 1948). So far, no functional pathway or genetic background was identified to enlighten its pathogenesis. Solely one pathological examination of a BSS-affected bull reports focal demyelination (Wells et al., 1987). In fact, for BSP, a decreased concentration of homovanillic acid in the cerebrospinal fluid was measured (De Ley and De Moor, 1975). Further, neuronal blocks of γ-efferent nerves by epidural administration of a 0.38% procaine solution interrupt clinical signs temporally (De Vlamynck et al., 2013, 2014). An RNA expression study of BSP in Romagnola cattle enforced the hypothesis of a neurodegenerative disease of glycinergic and dopaminergic pathways and showed significant differences in the expression of genes, which are known from hyperekplexia, amyotrophic lateral sclerosis (ALS), Parkinson's disease, and Huntington's disease in man (Pariset et al., 2013). However, none of these studies support BSP as a model for multiple sclerosis.

The aim of this study was the elucidation of the genetic background of hereditary spasms in high-yield sires and Holstein cattle population.

## Materials and Methods

### Ethical statement

EDTA blood samples of cattle were taken for routine parentage control exclusively by local veterinarians. Semen samples of routine production were collected from German artificial insemination centers. The collection of samples was approved by the Lower Saxony State Office for Consumer Protection and Food Safety (33.19-42502-05-17A196) according to §8a Abs. 1 Nr. 2 of the German Animal Protection Law.

### Clinical Investigations and Sample Collection

A total of 50 affected Holstein sires (16 sires born 1995–2001, 34 sires born 2010–2018) with signs of cramps of the lower limbs over a longer period were collected between January and April 2019. Complete pedigree data were provided by the Vereinigte Informationssysteme Tierhaltung (VIT) (Vereinigte Informationssysteme Tierhaltung, 2020). DNA was extracted from blood or semen samples using MagNa Pure LC DNA Isolation Kit I (Roche Diagnostics, Mannheim, Germany) or a modified salting out procedure (Miller et al., 1988). Pedigree analysis was performed using Pedigraph (Garbe and Da, 2003).

### Genome-Wide Association Study (GWAS)

Genotyping was performed using the BovineLD BeadChip and raw data were processed using GenomeStudio V2011.1 (Illumina, San Diego, USA). Final reports were imported into SVS version 8.8.3 (Golden Helix, Bozeman, MT, USA) to perform the association study.

Low-quality markers were filtered if call rates <0.95, minor allele frequency (MAF) <0.05, and carrier count <10. Only autosomes were analyzed. LD pruning was performed with default parameters (window size 50, increment 5, *r*^2^ threshold 0.5) reducing the initial 13,575 markers to 9,092. The window increment defines the number of markers by which the beginning window position will be incremented. In a broad context, a low window increment will provide enough of a pairwise comparison to remove those markers in high LD. However, if an increment value equal to the size of the window is picked, this will result in adjacent markers at the boundaries of the windows not being compared. This leaves some markers active that could be in high LD. Samples were selected by call rate >0.9. Identity by descent (IBD) was calculated and a maximum pairwise estimated PI of 0.46 was tolerated (Purcell et al., 2007).

SVS software denotes the probability that zero, one, or two alleles are identical by descent (“shared IBD”) by the notations P(Z = 0), P(Z = 1), and P(Z = 2), respectively. These probabilities may either refer to given markers or be thought of as sample-wide. SVS also uses a combined measure called PI, which is P(Z = 2) plus one-half of P(Z = 1). This is the probable number of shared alleles at any given marker.

PCA correction against the first three principal components was performed three times. Each time, eight principal components were calculated in the dataset and outlined samples were removed before PCA was calculated again. Therefore, the initial sample set of 577 samples (46 cases, 531 controls) was reduced to 259 samples (46 cases, 213 controls) (Patterson et al., 2006; Price et al., 2006). False positives due to the imbalance in cases/controls were initially accepted and later classified by results from further investigations. Genome-wide associations were calculated for all modes of inheritance as well as single-locus (SLMM) and multi-locus mixed models (MLMM) (Kang et al., 2010; Segura et al., 2012; Vilhjalmsson, 2012). Significance thresholds was considered as genome-wide 95% Bonferroni confidence interval (–log_10_-transformed *P* > 5.26). GWA study was designed as simple binary test not including any fixed effects.

### Identification of Candidate Variants by Whole Genome Resequencing

Ten samples were sequenced on a HiSeq2500 System (Illumina) resulting in ~10^9^ total reads per sample. Low quality (average phred quality <15) and single reads were removed resulting in ~9.4 × 10^8^ reads per sample. Final fastq files were aligned against bovine reference genome ARS-UCD1.2 with BWA-MEM, processed with Samtools v1.10 and resulting VCF files imported into SVS8.8.3 (Li and Durbin, 2009; Li, 2011; Shamimuzzaman et al., 2019; Rosen et al., 2020). Low-quality variant calls were filtered by genotype quality >0.8, read depth >10, and Alt read ratios (Ref_Ref <0.2, Ref_Alt inside 0.35 to 0.65 and Alt_Alt >0.8). This filtered out 28,111 from initial input 124,755 variant positions.

After QA steps, the remaining positions were called, if affected samples were homozygous carriers of the alternate allele and unaffected samples were heterozygous or homozygous wildtype. Annotated bovine genes were received from NCBI Bos taurus Annotation Release 106 (Pruitt et al., 2014). Finally, the remaining variants were filtered for their predicted effect on the amino acid sequence and only loss of function (LoF) and missense variants were further considered. SVS 8.8.3 identifies variants as “frameshift” if insertions or deletions within a transcribed (exonic) region are sized different than three bases.

Genotypes and allele frequencies of all candidate variants within the cattle cohort from run 8 of the 1000 Bull Genomes Project (Hayes and Daetwyler, 2019) were downloaded as VCF file and examined using BCFtools v.1.10.2 (http://wwwuser.gwdg.de/~falker/1kbulls/Run8/) (Li, 2011). This VCF file was aligned against bovine reference genome ARS-UCD1.2.

### Computational Models for Protein–Protein Interactions (PPI) and Protein Function

All transcribed genomic regions affected by a loss of function or missense mutation build a list of candidate genes. Computer models were created for the predicted interactions of candidate gene products from resequencing within networks of cramp-causing diseases, using Cytoscape v.3.8.0 and its StringApp utility for disease query (Shannon et al., 2003; Doncheva et al., 2019). Associations were accepted if String score >0.75. The modeled organism for all networks was *Bos taurus*. For associated proteins, the UniProt knowledgebase was examined (Uniprot Consortium, 2019). In addition, candidate genes were compared with publicly available databases for Mendelian diseases using keywords cramp, spastic paraplegia, hyperekplexia, amyotrophic lateral sclerosis, Parkinson, Huntington, and multiple sclerosis (Nicholas, 2003; Lenffer et al., 2006; Amberger et al., 2015; Bult et al., 2019; Smith et al., 2020).

For proteins where Cytoscape pointed out valid associations to known cramp-causing proteins, a String online search was conducted (Szklarczyk et al., 2019). Required interactions score was set to highest confidence (0.9) and network edges were set to “molecular action.” In the analysis tab, reference publications, and Reactome and KEGG Pathways were marked, if matching cramp-causing content (Kanehisa, 2019; Jassal et al., 2020).

The SWISS-Model online tool was used for homology modeling of predicted protein functions of prioritized candidate genes (Waterhouse et al., 2018). The primary amino acid sequences were used to model against 21 models from 387 templates found in the database. A QMEAN score of minimum −3.9 was used as quality threshold (Studer et al., 2020). For accepted models, 3D-structure images were created and colored by QMEAN values. Images to visualize location and effect of mutations within the gene model were created with Swiss-PdbViewer v.4.1 (Guex and Peitsch, 1997).

In addition, the primary amino acid sequence was used for an online BLAST search and alignment against several better examined species like human, mouse, rat, chimpanzee, rhesus macaque, and others (Kent, 2002). All high-quality alignments with >50% sequence identity and an E-value <1 × 10^−90^ were accepted and explored for experimentally validated expression data.

### Expression Analysis

For expression analysis, bovine liver, muscle, and brain RNA samples were used from a previous study (Zhang et al., 2019). RNA was converted to cDNA using High-Capacity cDNA Reverse Transcription Kit with RNase Inhibitor and dsDNase (ThermoFisher Scientific, Dreieich, Germany). Primer pair Ex43744.4_cDNA_F (5′-CTAAGAGAAATTTTGGTCCAT-3′) and LOC1008_cDNA2.0B_R (5′-CAGTCACTTTCTTTCTCCAG-3′) for LOC100848076 was designed. The amplicon generated with this primer pair gaped an intronic region and was therefore able to differentiate gDNA and cDNA. *GAPDH* (glyceraldehyde 3-phosphate dehydrogenase) and β*-actin* were used as internal controls. Amplification of *GAPDH* was performed using primers GAPDH_cDNA_fwd (5′-CCACTCCCAACGTGTCTGTT-3′) and GAPDH_cDNA_rev (5′-GCTTCACCACCTTCTTGATCTCATC-3′). For β*-actin* primers ACTB_cDNA_fwd (5′-GTCATCACCATCGGCAATGAG-3′) and ACTB_cDNA_rev (5′-AATGCCGCAGGATTCCATG-3′) were used (Huang et al., 2013; Macabelli et al., 2014). RNA concentrations and purity were measured on a NanoDrop (ThermoFisher Scientific). Resulting amplicons were separated on 2% agarose gels.

## Results

### Clinical Investigations and Pedigree Analysis

**Figure 1:**
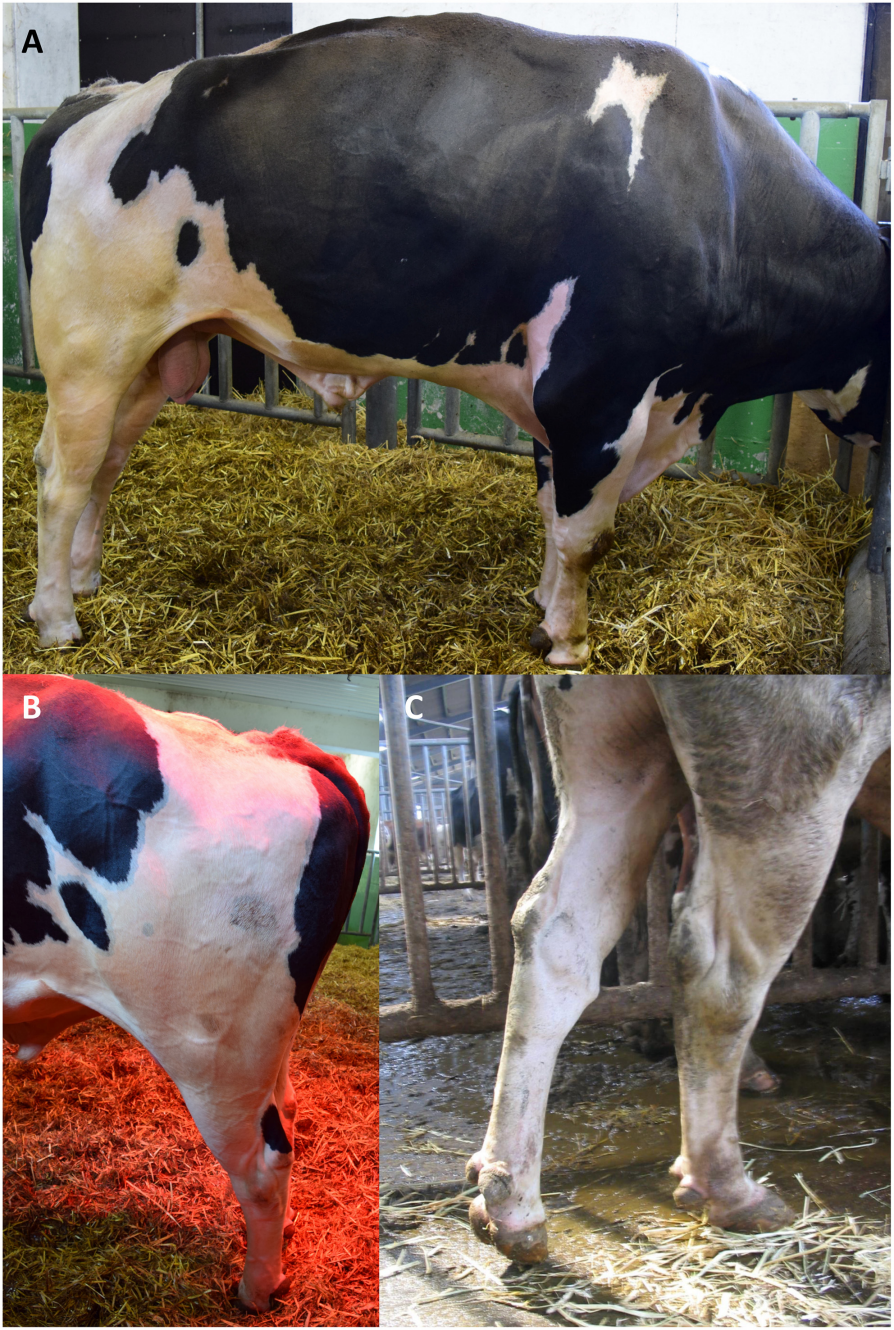
Clinical picture of BSS. **(A,B)** Four-year-old BSS-affected bull in a seizure. Mind the straightened hind legs and the straight hocks. The kyphosis is characteristic and helps to classify from BSP. The cramps last less than a minute and repeat multiple times. During seizures, the animal is immobilized. **(C)** Another bull with typical clinical sign of BSP. In this case, the cramp was a long-lasting event and appeared only unilateral. Straight hock was also present in BSP.

Clinical examination of the affected sires was performed at the artificial insemination centers. Affected sires were reported to show cramps in one or both hind legs, often accompanied by kyphosis (Figure 1). The majority of the animals were characterized by repeating seizures, with some having continuous cramps. These were classified as cases of late-onset BSP and also included in the study. The majority of affected animals presented with a nervous behavior while in standing position and showed an increasing tendency to lay down. Other behavioral abnormalities, as repeatedly feeding of small amounts of food were often reported, often combined with vocalization believed to indicate a reaction to pain or an expression of a rather complicated neuronal disease.

Pedigrees were constructed to identify common ancestors and to visualize the relatedness of the 50 affected sires. With this, it was possible to delineate a potential common founder (born in 1998) assumed to be a carrier of BSS due to its straight hocks. This bull was also commonly suspected to pass on BSP. The sire was an ancestor of 29 of the 50 affected sires, often appearing multiple times in the pedigrees and having also been used in many countries worldwide.

The latest common ancestor of all 50 sires was a bull born in 1965. However, this bull was also “founder” of the German Holstein population and is therefore present in most pedigrees only due to this fact. In addition, an 8-year-old healthy full sibling of an affected bull was identified and included in the analysis.

### Genome-Wide Association Study (GWAS)

**Figure 2:**
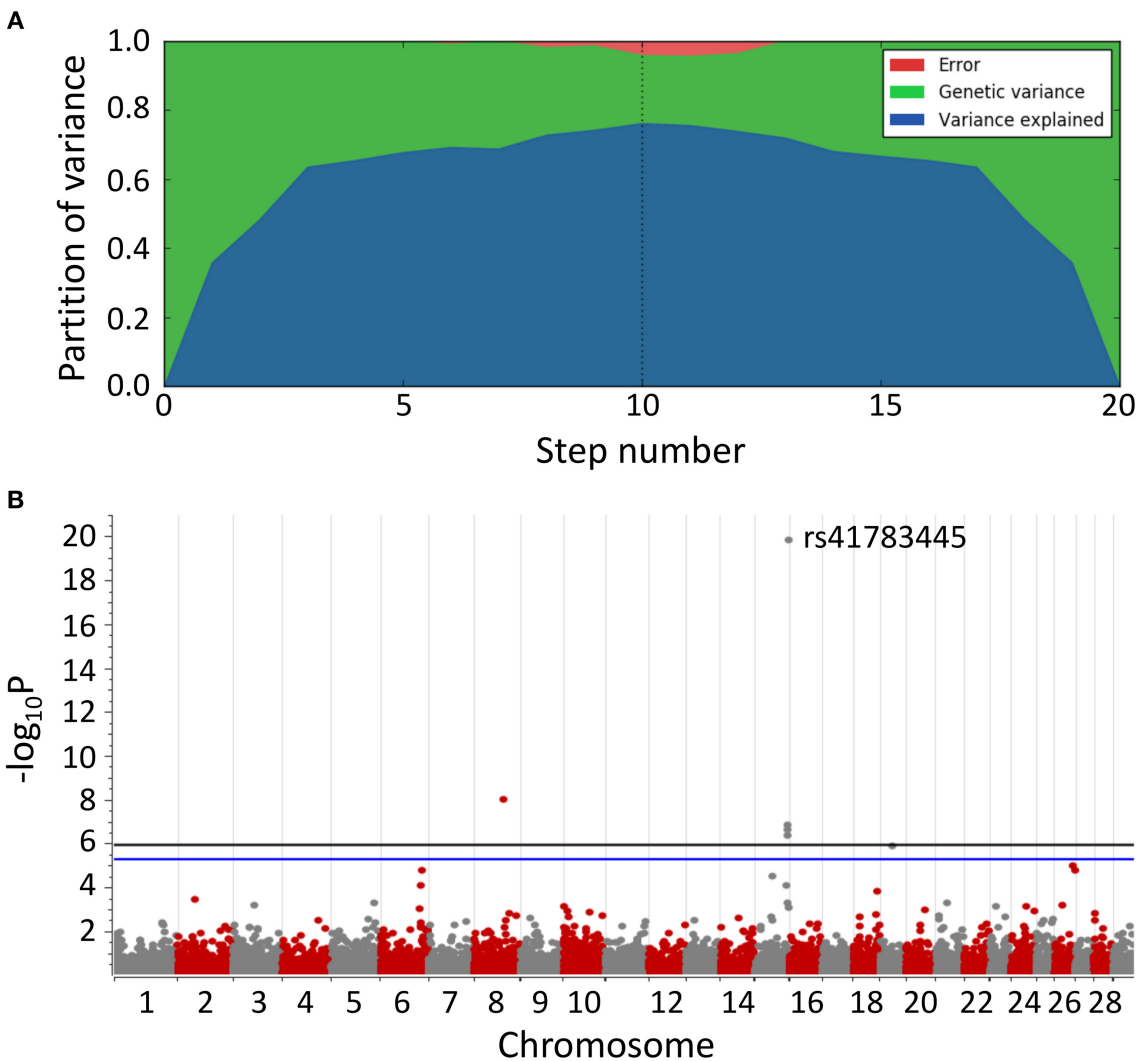
Manhattan plot of multi-locus mixed model (MLMM) for genome-wide associations for BSS/late-onset BSP. **(A)** Variance partition plot shows the model explained ~75% of the genetic variance. **(B)** Plotted are marker associations as negative log_10_-transformed *P*-values against the bovine chromosomal position (UMD3.1.1). Black line indicates the genome-wide 99.9% Bonferroni confidence threshold. Blue line indicates 95% Bonferroni confidence threshold. Associated genomic regions are on bovine chromosomes 8, 15, and 19.

After 10 steps, the MLMM explained about 75% of the genetic variance within the dataset. Plotting of the associations (*y*-axis, –log_10_P value against their chromosomal position (UMD3.1.1, *x*-axis) resulted in the Manhattan plot shown in Figure 2. All tested models (except recessive) highlighted a genomic region of ~1.6 Mbp on bovine chromosome 15 (BTA15) that spans the range 81,871,849–83,465,449 bp, represented by four loci on bovine chromosome 15 (BTA15), i.e., rs41783445, rs457101176, rs207681163, and rs384218732 exceeding the 95% Bonferroni-corrected significance threshold of –log_10_P = 5.26 (Table 1). Using the recessive model, two single markers on chromosomes 21 and 18 slightly exceed this threshold.

**Table 1:** Genotype frequencies and mixed linear model statistics (after step 1 of 10) from genome-wide association study highlighted markers rs41783445, rs457101176, rs207681163, and rs384218732 in BSS/late-onset BSP-affected and control cohort.

**Marker**	**rs41783445**			**rs457101176**			**rs207681163**			**rs384218732**		
**Genotype**	**C_C**	**C_T**	**T_T**	**C_C**	**C_G**	**G_G**	**A_A**	**A_T**	**T_T**	**A_A**	**A_G**	**G_G**
Cases	5	40	0	3	39	4	0	42	4	0	42	4
Controls	183	33	0	168	42	6	0	94	124	0	161	57
Total	188	73	0	171	81	10	0	136	128	0	203	61
HWE (χ^2^)^a^	6.89			0.01			31.78			102.99		
*P*-values (FET)^b^	1.43e^−21^			3.49e^−20^			6.99e^−10^			0.011		

a *HWE, Hardy–Weinberg equilibrium of total cohort*.

b *FET, Freeman–Halton extension of Fisher's exact test (non-directional two-tailed)*.

### Identification of Candidate Variants by Genome-Wide Resequencing

Based on the results of the pedigree analysis, 11 animals were selected for whole genome resequencing. These comprised one affected sire and his two full brothers (one known to be unaffected), two further affected sires and their respective parents, and the affected grandfather (mother's father) of one of these.

After QA steps, the remaining 206 homozygous alternate variants in affected sires (while heterozygous in unaffected) were filtered for their predicted effect on the amino acid sequence and only loss of function (LoF) and missense variants were further considered. All detected variants were insertions.

With this approach, eight variants were identified in eight different genes, i.e., seven variants causing a loss of function due to frameshifts within exonic regions and one missense variant creating a premature start codon (Table 2).

**Table 2:** Functional variants detected by whole genome resequencing.

**BTA^a^**	**Position^b^**	**Sequence ontology^c^**	**Region**	**Effect^d^**	**Gene name**	**Exon number**	**Number of exons**	**HGVS g.**
6	117117723	FSV	Exon	LoF	UVSSA	1	13	NC_037333.1: g.117117722_117117723insA
15	51379526	FSV	Exon	LoF	LOC112441636	1	4	NC_037342.1: g.51379525_51379526insA
15	51402894	FSV	Exon	LoF	LOC407145	4	6	NC_037342.1: g.51402893_51402894insG
15	78135823	FSV	Exon	LoF	LOC100848076	3	3	NC_037342.1: g.78135822_78135823insC
18	49991574	FSV	Exon	LoF	COQ8B	5	16	NC_037345.1: g.49991573_49991574insC
22	1645924	FSV	Exon	LoF	NEK10	19	38	NC_037349.1: g.1645923_1645924insA
28	2318868	FSV	Exon	LoF	PGBD5	7	8	NC_037355.1: g.2318867_2318868insA
29	28264530	SCG	5UTR	Missense	MSANTD2	1	6	NC_037356.1: g.28264529_28264530 insAAGAAGCAGGAGGCAGGCATGAATCAAGT

a *BTA, Bos taurus chromosome*.

b *Positions according to reference genome ARS-UCD1.2*.

c *FSV: frameshift variant, SCG: 5′-UTR premature start codon gain variant*.

d *LoF: loss of function*.

Accepting heterozygous and homozygous wildtype in the unaffected full sibling resulted in 13 further variants, but on highlighted chromosome 15, no additional variant was listed. Also, none of these additional variants were highlighted by any further method mentioned later.

Variant NC_037342.1:g.78135822_78135823insC in LOC100848076 was located directly within the first codon of exon 3 (of 3) causing a frameshift. In addition, this variant is located within the strongly associated genetic region on BTA15 by the GWA study. The other variants listed in Table 2 do not match a genomic region identified by the GWAS. GWAS and whole genome resequencing were executed on different reference genomes (UMD3.1.1 and ARS-UCD1.2). Using reference genome UMD3.1, the location of LOC100848076 (i.e., ENSBTAG00000030909) was on chromosome 15:79346444-79347350.

### Prioritization of Candidate Variants Using Databases and Protein–Protein Interaction Networks (PPI)

None of the candidate genes listed in Table 2 appeared within the databases for Mendelian inheritance in connection with the mentioned keywords.

**Figure 3:**
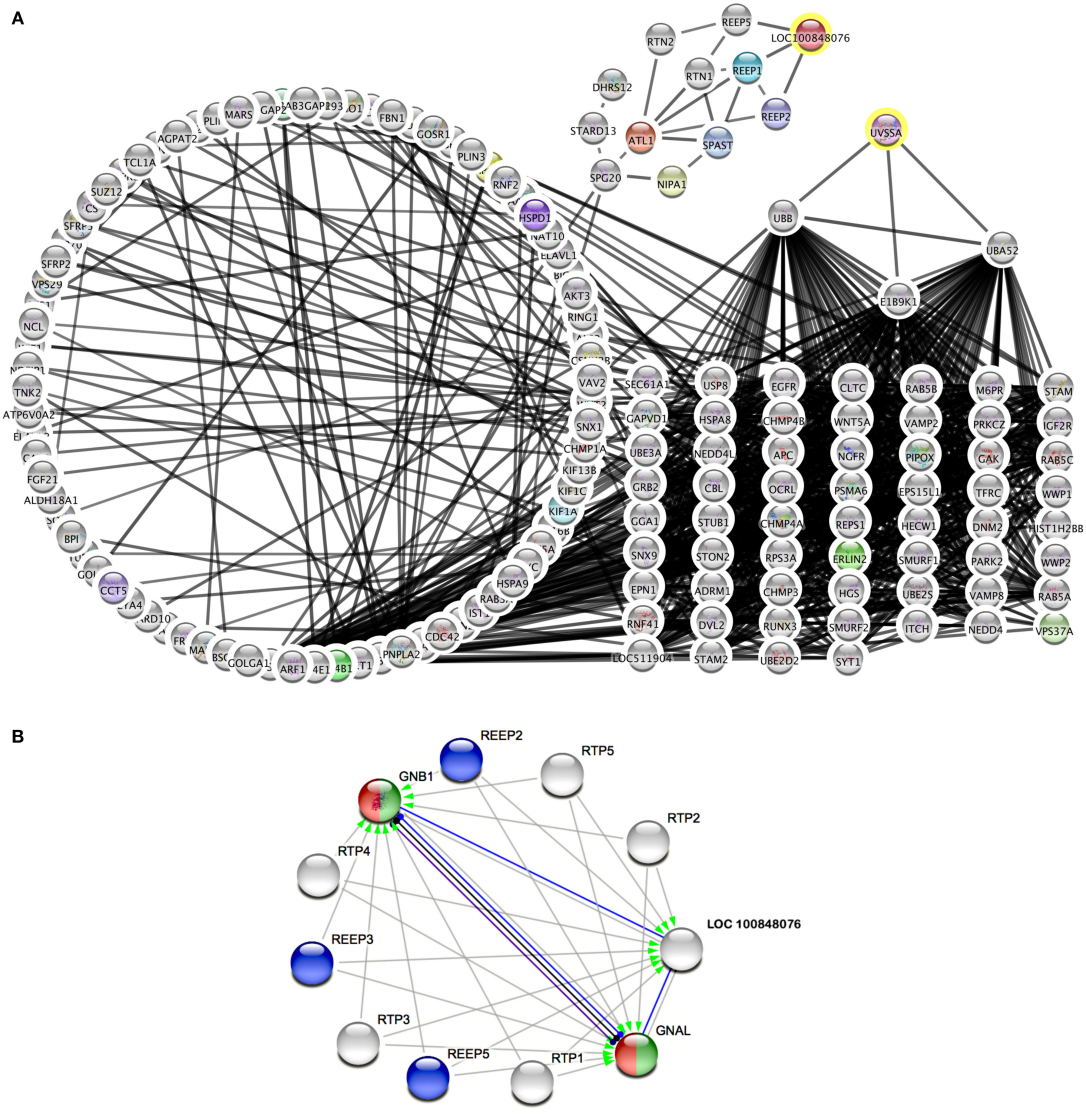
Protein interaction networks of candidate gene products. **(A)** Merged network of String disease network for Troyer syndrome and products from BSS/late-onset BSP candidate genes with 0.75 String confidence edges. Candidate genes marked with yellow rings. **(B)** Image from String online search (String Consortium, 2020) for LOC 10084076 with highest confidence (0.9) edges. Gray arrows symbolize activation, blue edges symbolize binding, black edges symbolize reaction, and purple edges symbolize catalysis. Green color marks “Activation of GABA-B-Receptor” and red color marks “Opioid Signaling” from Reactome Pathways. REEP proteins from **(A)** are colored blue.

Therefore, metabolic pathways were searched to link known genes for cramp-causing diseases. Within Cytoscape, the StringApp disease query was applied to import validated networks of diseases that match BSS symptoms. To connect the candidate genes to any low-degree nodes within a 360-protein network for multiple sclerosis failed even when the String confidence threshold was reduced to a medium level (0.4). This approach was retried within a 313-protein network for Troyer syndrome, a human disease also known as human spastic paraplegia 20 (HSP20) and here high confidence interactions (0.75) of two candidate genes UV Stimulated Scaffold Protein A (*UVSSA*) and LOC100848076 to gene products in the disease network were identified (Figure 3). The interactions of UVSSA were linked to high-degree nodes, representing genes that were multiply connected and therefore believed to be false positives.

LOC100848076 was connected to low-degree nodes, representing the Receptor expression-enhancing proteins (REEP) 1, 2, and 5. These connections were even of highest confidence (0.9). Some of these proteins are causative for spastic paraplegias in human. Further, spartin (*SPG20*), spastin (*SPAST*), atlastin (*ATL1*), non-imprinted in Prader-Willi/Angelman syndrome-1 (*NIPA1*), and reticulon-2 (*RTN2*) are strongly connected to these proteins. Except of REEP5, all these proteins are causative for different forms of spastic paraplegias in humans.

Using String online search unique for LOC10084876 (Figure 3B), strongest interactions (String confidence 0.9) in a small network were outlined, containing receptor expression-enhancing proteins (REEP), receptor-transporting proteins (RTP), and additionally guanine nucleotide binding protein, alpha activating activity polypeptide, olfactory type (GNAL), and guanine nucleotide-binding protein, eta-1 (GNB1).

### Exploration of LOC100848076 by Homology Modeling

**Figure 4:**
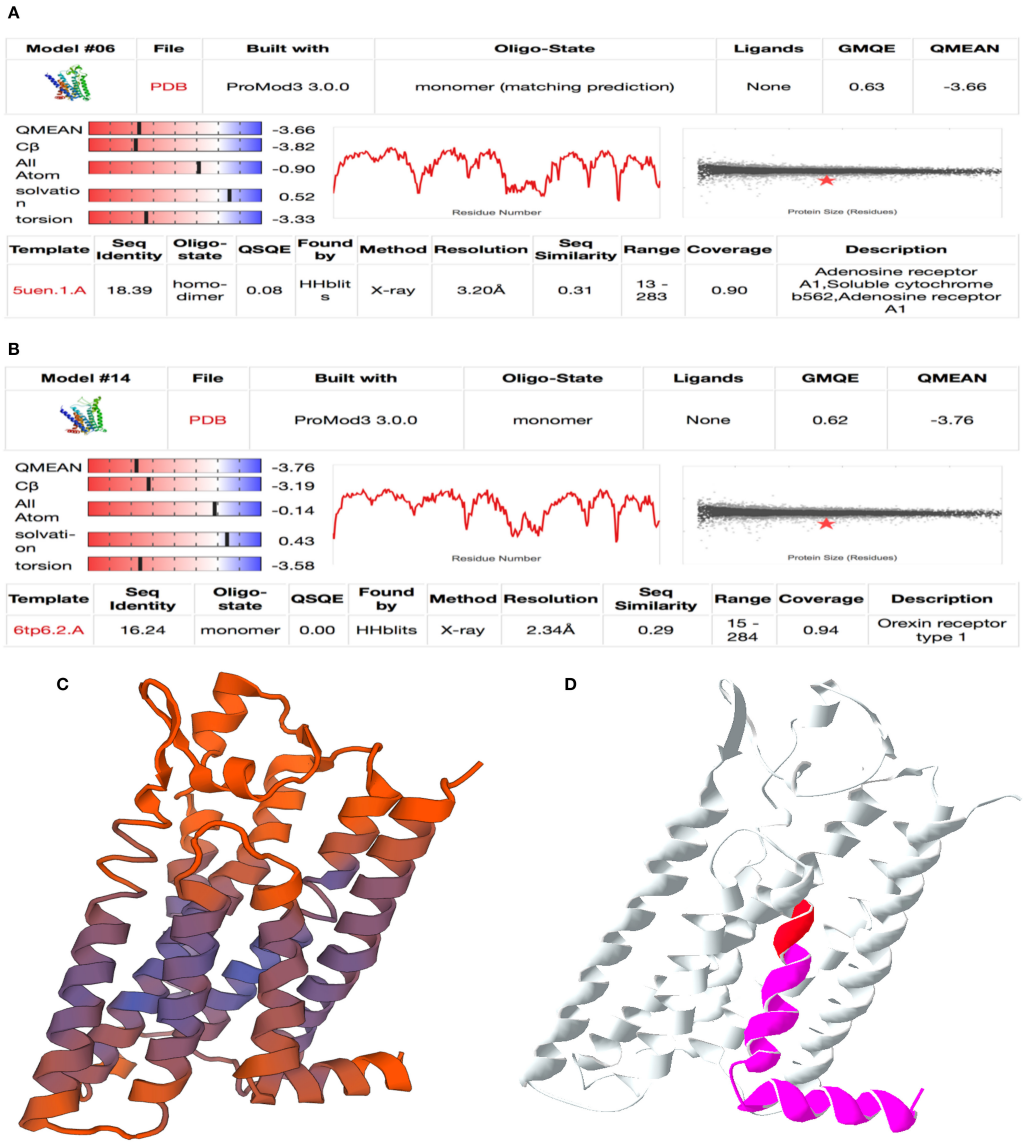
Result of homology modeling for LOC100848076 with SWISS-MODEL online tool (Swiss Institute of Bioinformatics, 2020). **(A,B)** Summary table of model fitting for **(A)** adenosine A1 receptor and **(B)** orexin receptor type 1. Mind the QMEAN (Qualitative Model Energy ANalysis), GMQE (Global Model Quality Estimation), QSQE (Quaternary Structure Quality Estimation), and sequence identity values are relevant for model selection. **(C)** QMEAN-colored cartoon model for adenosine A1 receptor. Purple color shows well-fitting areas and red color shows minor quality sequences. **(D)** Mutation cartoon model of adenosine-A1-receptor model. Red area marks the location of the insertion NC_037342.1:g.78135822_78135823insC and magenta area marks the frameshift affected protein sequence downstream.

The two proteins adjusted in SWISS model were adenosine receptor A1, soluble cytochrome b562, adenosine receptor A1 (QMEAN −3.66), and orexin receptor type 1 (QMEAN −3.76) (Figure 4). An image demonstrating the goodness of fit for the adenosine receptor A1 is shown in Figure 4C. Both proteins modeled are G protein-coupled receptors.

Considering the clinical signs of the affected sires, orexin receptor was a badly fitting model because it is mainly affecting hunger and sleeping behavior (Rappas et al., 2020). In contrast, adenosine A1 receptor obviously identified as a well-fitting model. It is relevant for neuronal signal transduction in striatal medium spiny neurons. Adenosine A1 receptors are found to be expressed in several tissues. The identified frameshift mutation modifies the last of the seven transmembrane domains and the intracytoplasmic α-helix domain at the C terminus (Figure 4D).

### Determination of Candidate Variant Frequency Within the 1000 Bulls Project

Alternative-allele frequencies ranged from 22.3 to 84.8% and are therefore assumed to represent rather common variants within the worldwide cattle population. The frequency of the presumed most likely causative variant g.78135822_78135823insC within LOC100848076 was 73.7%, and in a subset of 700 Holstein cattle, 436 homozygous carriers of alternative allele were detected.

### Expression Analysis of LOC100848076 in Bovine Liver, Muscle, and Brain

So far it was not possible to detect a transcript of LOC100848076 in randomly selected bovine RNA samples from liver, muscle, and brain. Nonetheless, expression of two homologs of LOC100848076 with at least 50% amino acid sequence identity in mouse and six homologs in human has been experimentally proven (Tsuboi et al., 1999; Taylor et al., 2006; Zhang et al., 2007; Tian and Ma, 2008; Duan et al., 2012; Uniprot Consortium, 2019). Three of these are expressed in neuronal tissue, thereof one (*OR4C16*) in sural nerve.

## Discussion

Bovine spastic syndrome (BSS) and late-onset bovine spastic paresis (BSP) are heritable diseases causing severe pain to affected animals and proceed chronic-progressively. Therefore, the prognosis for affected animals is most unfavorable. Notifications increase that inherited spasms emerge in accommodated sires at artificial insemination centers (Gentile and Testoni, 2006; Goeckmann et al., 2018). Thus, the underlying genetic condition is spread far over cattle populations and relevance for animal welfare and economics is continuously growing. The prevalence for BSS is frequently assumed to be <5% (Goeckmann et al., 2018) and for late-onset BSP lower than 1% (Goeckmann et al., 2016). However, quite obviously, these frequencies are underestimated due to the late onset of the disease symptoms and the noticeably decreased life span most cattle experience in today's dairy production. Furthermore, combining the likely facts that these diseases are found frequently in breeding sires and these traits are inherited, the actual prevalence must be clearly elevated. Within the analyzed cohort, sires were found showing clearly separable clinical pictures for either BSS or BSP (either continuous cramps or repeating seizures), even if genetic background seems uniform in this study. Based on this, there is strong likelihood for BSS and late-onset BSP being different manifestations on an identical genetic background.

To separate sires in this case cohort from seizures of a non-genetic determination, animals were only selected from artificial insemination centers. These sires are daily supervised by veterinarians and correct assessment of duration and quality of the cramps is guaranteed. However, several environmental and animal husbandry factors potentially causing spasms in cattle need to be taken into consideration. Goeckmann et al. separate these in nutritional factors, environmental factors, individual factors, and other pathogenic agents (Goeckmann et al., 2016). They point out plant and mycotoxin intoxication, manganese and other trace element shortages, vitamin A or C deficiencies, season and mothers' condition during birth, sex, sarcosporidiosis, encephalitis, and also bovine spongiform encephalopathy. Herpesvirus infections are furthermore discussed as causative for pathological motion in multiple sclerosis in humans (Banwell et al., 2007). The most important factors assumed by the authors of this study are intoxications, lack of manganese, hypovitaminoses, and also hypocalcemia, which is a common shortage in dairy cattle. Nonetheless, nearly all of these factors are expected under control in a herd of genetic-valuable sires.

In the present study, a significantly associated variant NC_037342.1:g.78135822_78135823insC on BTA15 was identified. This variant causes a loss of function in a predicted G protein-coupled receptor and possible adenosine-A1-receptor homolog (LOC100848076) due to a frameshift. This frameshift affects the seventh transmembrane domain and the downstream cytoplasmic C-terminal α-helical domain. Especially this domain is essential for correct installation and fixation of the receptor within the cell membrane. It additionally acts as an important contact domain for many receptor-associated proteins (Bockaert et al., 2003). The frameshift mutation likely decreases the receptor activity and possibly inhibits secondary messenger systems related to this G protein-coupled receptor by inability to interact.

There was no possibility to specify the mode of inheritance or penetrance within this cohort because ancestors' phenotypes were often not reported. Two possible genetic explanations are envisaged for the discrepancy between the found allele frequency and the observed disease prevalence in the field, i.e., either a multigenic inheritance or a gene–environment interaction. Within a cohort of 3,093 worldwide and multibreed animals, variant NC_037342.1:g.78135822_78135823insC had an allele frequency of nearly 74%. Even if inherited spasms are common in breeding bulls and massively underestimated, due to the late onset, this is very unlikely to be the only explanation for it. Most likely, the mode of inheritance is polygenic. First, polygenic inheritance could be an explanation for the observed low prevalence of BSS and late-onset BSP and second for the often reported incomplete penetrance (Goeckmann et al., 2016). Therefore, an autosomal (probably recessive) multigenic inheritance for BSS/late-onset BSP is supported.

Whole genome resequencing did not reveal a sex chromosome–linked variant. However, in the GWA study, further associated chromosomal regions were present on chromosomes 8 and 19. By using additive, dominant, or genotypic model as well as a basic allelic test additionally, a genomic region on bovine chromosome 23 was associated by two genetic markers. Within the strongly associated region on BTA15, only the outlined variant explained the biological background of cramps due to whole genome resequencing. Indeed, assuming the healthy full sibling was heterozygous at this position, a recessive, multigenic inheritance is still possible. Accepting homozygous alternative alleles also in the healthy full sibling, 12 loss-of-function and 2 missense variants were found within the GWAS-associated chromosomes 8, 15, 19, and 23. Two of these 14 affected genes (OR51I1 on BTA15 and STN1 on BTA23) have also strong linkage to receptor expression-enhancing proteins (REEP). The GWA study did not support the associated regions on BTA7 and 9 described earlier (Neustaeter et al., 2015).

The present study indicated that inherited cramps in cattle could be diseases of GABAergic and dopaminergic metabolic pathways. Presumably, LOC10084876 plays a role in the direct dopamine pathway (Glukhova et al., 2017; Stockwell et al., 2017). That agrees with the accepted neuronal background for BSP and BSS (Gentile and Testoni, 2006; Pariset et al., 2013; De Vlamynck et al., 2014; Goeckmann et al., 2016, 2018). Assuming adenosine-A1-receptor activity for LOC10084876, the supposed affected cell type could be the medium spiny neuron (D1-type) (direct pathway MSN) of the dorsal corpus striatum in the forebrain. Striatum is an important structure of the motor system (Fuchs et al., 2013). Here, GNAL and GNB1 act as the alpha- and beta-subunits of a heterotrimeric G-protein, thus relegating signaling after activation of transmembrane receptors (Fuchs et al., 2013). Their activation is possibly disabled by the frameshift mutation, if these G proteins correspond with the receptor encoded by LOC10084876.

MSNs are GABAergic neurons, acting excitative, if adenosine A1 receptor and D1-type dopamine receptor are expressed, or inhibitory, if adenosine A2 receptor and D2-type dopamine receptor are expressed (Herve et al., 1993; Fuchs et al., 2013). Direct dopamine pathway MSNs physiologically inhibit pallidum and substantia nigra by GABA release, and these structures finally control motoneurons in the pyramidal system by inhibition itself. In D1-type MSNs, after activation of adenosine A1 receptors, the release of synaptic vesicles decreases by inhibition of presynaptic Ca^2+^ channels (Fredholm et al., 2011) and this controls GABA release. This receptor function can be assumed to be lost in LOC100848076 homozygous carriers. The alterations cause disequilibrium of direct and indirect pathways which result in Parkinson-type pathological motor control (Yager et al., 2015; Parker et al., 2016). According to that, spasms in cattle resulting from degeneration of the direct dopamine pathway seem obvious. Chorea Huntington is another modeled disease in this context because it results from degenerations in the indirect dopaminergic pathway (Roze et al., 2010).

The protein interaction models build reference to the human disease complex of hereditary spastic paraplegias (HSPs) and possibly Parkinson's disease (Jaberi et al., 2016), rather than to multiple sclerosis, in addition to primary torsion dystonia by interaction to *GNAL* (Fuchs et al., 2013) and to severe hypotonia by *GNB1* (Petrovski et al., 2016). Both diseases are also causing seizures, even if their clinical picture is much more severe than in most HSPs. However, one study reports milder clinical pictures of HSPs more often observed in male patients (Schule et al., 2016).

It is conceivable that LOC10084876 might interact with *REEP1, 2*, or *5*. Mutations in *REEP1* are causative for human autosomal dominant spastic paraplegia 31 (Schlang et al., 2008; Richard et al., 2017) and mutations in *REEP2* are causative for spastic paraplegia 72 in humans (Esteves et al., 2014). *REEP5* has not yet been associated to any cramp-causing disease, but is functionally relevant (like all REEP proteins) for shaping of the tubular endoplasmic reticulum, therefore influencing the transport of G protein-coupled receptors to the cell surface membrane (Voeltz et al., 2006). This is a frequently reported cellular mechanism within HSP (Boutry et al., 2019). In addition, consanguinity is very often described in families affected by HSP (Novarino et al., 2014; Bizzari et al., 2017) and also present in the analyzed animal cohort. Within the Cytoscape network, other causative genes for HSP were strongly connected to *REEP1, REEP2*, and *REEP5*. These were spartin (*SPG20*), spastin (*SPAST*), atlastin (*ATL1*), non-imprinted in Prader-Willi/Angelman syndrome-1 (*NIPA1*), and reticulon-2 (*RTN2*), causative for Troyer syndrome, autosomal dominant spastic paraplegia 4, autosomal dominant spastic paraplegia 3A, autosomal dominant spastic paraplegia 6, and autosomal dominant spastic paraplegia 12, respectively (Reed et al., 2005; Shoukier et al., 2009; Montenegro et al., 2012; Varga et al., 2013; Bizzari et al., 2017).

One of these HSP-relevant cellular pathways, either ER shaping or loss of neuronal signaling transduction, could also be altered in BSS/late-onset BSP. ER shaping means an exchange in the interactions between endoplasmic reticulum and cytoskeletal structures, especially within the neuronal axon. Physiological ER shaping is necessary for the transport of membrane-linked proteins to the neuron's periphery after protein biosynthesis. Essential for ER shaping are receptor expression-enhancing proteins (REEP) as well as spastin and atlastin. Alterations in ER shaping are causative in multiple HSP and also a pathological feature in Alzheimer's disease (Sharoar et al., 2016). Even if none of these genes were altered in the investigated sires, this additionally links to a membrane-located protein. LOC100848076 was identified as a G protein-coupled adenosine receptor and therefore could better fit the reduced neuronal signaling hypothesis.

Pariset et al. also mentioned Morbus Parkinson, Chorea Huntington, and other human diseases after RNA expression analyses in BSP-affected animals (Pariset et al., 2013) and parkinsonism was already described in forms of HSP (Novarino et al., 2014; Boutry et al., 2019). Hyperekplexia in humans, a disease likewise mentioned by Pariset et al., was identified to cause altered gamma aminobutyric acid concentration in cerebrospinal fluid (Dubowitz et al., 1992). This corresponds to the direct dopamine pathway hypothesis mentioned before in this study.

Regarding LOC100848076, no animal models or human disease has been reported so far. In one BSS case report, demyelination was detected, which was later interpreted as a link to multiple sclerosis (Wells et al., 1987; Goeckmann et al., 2018). However, similar lesions were later identified also in Charolais cattle with progressive ataxia and American Brown Swiss with spinal demyelination (Thomsen et al., 2010; Duchesne et al., 2018). Spinal demyelination is caused by *SPAST* and was already mentioned for HSP4 before (Shoukier et al., 2009). Progressive ataxia is a disease caused by an alteration in the gene Kinesin Family Member 1C (*KIF1C*). Spastic ataxia in humans is also caused by this gene, but other genes from the kinesin family member (KIF) family are known to cause HSP10 and 30 (Erlich et al., 2011; Novarino et al., 2014; Carosi et al., 2015). *KIF* genes encode for kinesins, relevant for protein transport along cytoskeletal structures like microtubules. No further pathological findings were described so far in the literature for BSS and BSP. Clinical picture in cattle affected by spinal demyelination or progressive ataxia are very similar to those observed in this cohort. Thus, cellular pathways using *SPAST* or *KIF1C* are also likely causative in BSS/late-onset BSP in Holstein cattle and both are already known to be relevant in HSP.

The genetic background of BSS/late-onset BSP seems to affect the brain's dopamine or GABA metabolism. Several therapeutics for HSP or other spasms target these pathways, like Baclofen, botulinum toxin, benzodiazepines, and others (Casari and Marconi, 2006; Schwenk et al., 2010; Geng et al., 2013; Pariset et al., 2013; Van Lith et al., 2019). Unfortunately, due to the German (and maybe all international) drug law, none of these drugs is authorized for food-producing animals. Therefore indeed, no novel drugs are available to reduce pain in affected cattle based on the results of this study. Nonetheless, results of this study can be used for a breeding approach to reduce the appearance of inherited cramps in cattle and as a disease model for the mentioned human diseases. But hitherto, no diagnostic test was applied due to left uncertainties because of unexpected high allele frequency.

In this study, GWAS, family-based NGS analysis, and protein–protein interaction network modeling independently highlighted LOC10084876 as a potential genetic reason for Holstein cattle to develop BSS/late-onset BSP. The gene product likely encodes a G protein-coupled receptor. An insertion (NC_037342.1: g.78135822_78135823insC), common in the investigated 50 sire cohort, caused a frameshift affecting the last transmembrane domain and the intracytoplasmic C-terminal α-helical domain. Most likely, this causes a decrease or loss of neuronal transmitter release and affects a cellular pathway, also relevant for certain HSP in man.

## Data Availability Statement

The datasets presented in this study can be found in online repositories. The names of the repository/repositories and accession number(s) can be found at: www.osf.io/yw52d, BSP_files.bed; www.osf.io/yw52d, BSP_files.bim; www.osf.io/yw52d, BSP_files.fam.

## Ethics Statement

The animal study was reviewed and approved by Lower Saxony State Office for Consumer Protection and Food Safety (33.19-42502-05-17A196). Written informed consent was obtained from the owners for the participation of their animals in this study.

## Author's Note

During the review process, expression of LOC1008480076 transcript XM_015474960.2 was experimentally proven by RT-PCR in bovine brain, skeletal muscle, liver and eight additional tissues.

## Author Contributions

FK performed the experiments and analyzed the data. BB conceived the project and performed genotyping, sequencing, and data analysis. FK, MH, and BB wrote the manuscript. WW performed pedigree analysis and sample acquisition. All authors contributed to the article and approved the submitted version.

## Conflict of Interest

The authors declare that the research was conducted in the absence of any commercial or financial relationships that could be construed as a potential conflict of interest.
